# Bioavailability, Metabolism, and Excretion of [^14^C]‐Tazemetostat in Patients With B‐Cell Lymphomas or Advanced Solid Tumors

**DOI:** 10.1002/cpdd.1508

**Published:** 2025-02-03

**Authors:** Yingxue Chen, Renli Teng, Julien Ogier

**Affiliations:** ^1^ Clinical Pharmacology, DMPK and Pharmacometrics Ipsen Bioscience Inc. Cambridge MA USA; ^2^ CareCeutics, LLC Berwyn PA USA; ^3^ Clinical Pharmacology, DMPK and Pharmacometrics Ipsen Innovation Les Ulis France

**Keywords:** bioavailability, drug excretion, drug metabolism, pharmacokinetics, tazemetostat

## Abstract

This open‐label, multicenter study (NCT03010982) evaluated the absolute bioavailability, characterized the disposition and metabolism, and investigated the metabolic profile of tazemetostat, a US Food and Drug Administration–approved inhibitor of enhancer of zeste homolog 2, following intravenous and oral [^14^C]‐labeled and unlabeled tazemetostat in patients with B‐cell lymphomas or advanced solid tumors. Patients received oral tazemetostat 800 mg twice daily for 14 days. On Day 15, patients received tazemetostat 800‐mg tablets in a fasted state followed by an intravenous microdose of 12 µg [^14^C]‐tazemetostat. On Day 16, patients received a [^14^C]‐tazemetostat 800‐mg solution with a meal, then continued tazemetostat 800 mg twice daily. Blood, plasma, urine, and fecal samples were collected for pharmacokinetic analyses, and recovery and excretion of the radioactivity of [^14^C]‐labeled/unlabeled tazemetostat and its metabolites. The median absolute bioavailability was 31.8% (range, 20.2%‐49.8%). Notable plasma components were EPZ‐6930, unchanged tazemetostat, EPZ006931, and EPZ034163, accounting for 31.8%, 22.4%, 11.0%, and 3.5% of total drug‐related exposure, respectively. Recovery of radiolabeled material ranged from 93.2% to 94.7%, with most excreted doses recovered within 48 hours in urine and by 96 hours in feces. Fecal elimination represented the principal route of elimination with a mean of 78.9% of the administered radioactive dose and renal excretion accounted for 15.4%.

Epigenetic regulation is a genomic process that reversibly modifies gene expression without altering the underlying DNA sequence. Dysregulation of epigenetic pathways has been observed in some cancer types. Examples of epigenetic abnormalities include aberrant histone modifications that can lead to the activation of oncogenes or inhibition of tumor‐suppressor genes.^1^, ^2^ As a result, there is a high level of interest in targeting components of epigenetic pathways for antitumor therapy.^3^, ^4^

Enhancer of zeste homolog 2 (EZH2) is a histone methyltransferase and an epigenetic regulator of cellular differentiation.^5^ EZH2 is the catalytic subunit of the polycomb repressive complex 2 that is responsible for the trimethylation of histone 3 lysine 27 (H3K27me3), an important process for genetic repression and cell‐cycle regulation.^1^, ^5^ EZH2 controls the formation and function of the germinal center (GC) and is involved in B‐cell differentiation.^6^ Following their initial activation, B cells enter the GC and upregulate EZH2. This represses gene expression that would otherwise limit B‐cell proliferation, promote differentiation, and cause B cells to exit the GC.^6^, ^7^, ^8^ Instead, B cells remain in the GC, allowing them to proliferate with the potential for malignant transformation.^7^, ^8^, ^9^ When EZH2 activity is downregulated upon B‐cell exit of the GC, reversing H3K27me3‐mediated gene repression, B cells are normally able to form part of the humoral immune response through replication and differentiation into memory or plasma cells, the formation of high‐affinity antibodies and B‐cell receptors, or otherwise undergo apoptosis.^6^, ^9^ EZH2 is overexpressed in both solid and hematologic malignancies and its overexpression is associated with a poor prognosis, making EZH2 a desirable therapeutic target.^1^, ^8^

Tazemetostat is a first‐in‐class oral inhibitor of EZH2 approved by the US Food and Drug Administration for patients with follicular lymphoma and epithelioid sarcoma.^10^, ^11^ Tazemetostat received accelerated approval based on the clinical activity demonstrated in 2 open‐label Phase 2 trials; NCT01897571 evaluated tazemetostat for patients with advanced follicular lymphoma, and NCT02601950 enrolled patients with epithelioid sarcoma.^8^, ^10^, ^12^

The pharmacokinetic (PK) properties of tazemetostat have been previously described.^13^ The systemic exposure of tazemetostat is approximately dose proportional for doses ranging from 200 to 1600 mg twice daily. Steady state was reached by Day 15 following oral administration of tazemetostat 800 mg twice daily.^13^ Tazemetostat exhibited time‐dependent PK, with a mean accumulation ratio (measured by the area under the concentration–time curve [AUC]) of 0.58. Tazemetostat is absorbed rapidly, reaching a maximum observed plasma concentration (C_max_) in 1‐2 hours, with 88% of tazemetostat bound to human plasma proteins in vitro.^10^, ^13^ Current evidence suggests that tazemetostat absorption is not affected by food intake; patients given tazemetostat 200 mg with a high‐fat, high‐calorie meal (approximately 800‐1000 calories) did not experience any significant changes in tazemetostat exposure compared with those given tazemetostat fasted.^14^ In vitro, tazemetostat is metabolized by cytochrome P450 (CYP) 3A to form inactive metabolites, EPZ‐6930 and EPZ006931, with EPZ‐6930 being further metabolized by CYP3A.^10^

The objective of this open‐label, multicenter study (NCT03010982) was to determine the absolute bioavailability, characterize the disposition and metabolism, and investigate the metabolic profile of tazemetostat following administration of intravenous and oral [^14^C]‐labeled and unlabeled tazemetostat in patients with B‐cell lymphomas or advanced solid tumors, as such studies are required by regulatory authorities.

## Methods and Materials

### Study Design

**Figure 1:**
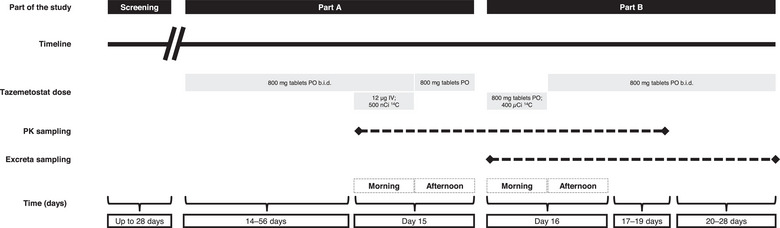
Study design. b.i.d., twice daily; IV, intravenous; PK, pharmacokinetics; PO, oral administration.

The study design is represented in Figure 1. Patients were screened for up to 28 days before the start of tazemetostat treatment and safety assessments. Patients were admitted to the hospital the day before the first dose of [^14^C]‐labeled tazemetostat and remained in the hospital until 5 days after the oral dose of [^14^C]‐tazemetostat or earlier if 90% or more of the administered radioactive dose had been recovered or less than 1% of the total dose in a 24‐hour collection of urine and feces had been demonstrated.

In Part A, patients received oral tazemetostat 800 mg twice daily for a minimum of 14 days and a maximum of 56 days with the aim of characterizing absorption, disposition, metabolism, and elimination at steady‐state concentrations. On the morning of Day 15, patients received tazemetostat 800 mg as tablets in a fasted state followed by a 12‐µg microdose of [^14^C]‐labeled tazemetostat containing 500 nCi of [^14^C]‐tazemetostat (the structure of [^14^C]‐tazemetostat is shown in Figure S1) administered as an intravenous bolus over 30 seconds in a fasted state, starting 1 hour after the oral dose. Tazemetostat microdosing was selected for this study to better understand tazemetostat PK without requiring a large patient cohort or increasing the burden on patients.

In Part B, on the morning of Day 16, patients received an oral dose of tazemetostat 800 mg containing approximately 400 µCi of [^14^C]‐tazemetostat as a solution with a meal. After approximately 12 hours, patients resumed tazemetostat 800‐mg twice‐daily tablets.

After completing all study assessments for both parts of the study and at least 20 days of tazemetostat dosing, patients who were benefitting from treatment were permitted to continue treatment with tazemetostat for up to 6 cycles or 24 weeks.

The study was conducted across 2 study sites in the United Kingdom: Royal Liverpool and Broadgreen University Hospital Trust (Liverpool, United Kingdom) and The Clatterbridge Cancer Centre NHS Foundation Trust (Bebington, United Kingdom).

The study was conducted and reported in accordance with the Declaration of Helsinki, Good Clinical Practice as described in the International Conference on Harmonisation Tripartite Guideline E6, and local legal and regulatory requirements. Relevant study documents, including the protocol, were approved by the Independent Ethics Committee, Health Research Authority (Manchester, United Kingdom), for each center before initiation of the study. All patients provided written informed consent.

### Eligibility Criteria

Eligible patients were adults aged 18 years or above with histologically confirmed relapsed or refractory B‐cell lymphomas who had received at least 2 prior lines of therapy, or histologically or cytologically confirmed advanced or metastatic solid tumors that had progressed after treatment with approved therapies or for whom there were no standard therapies available. Patients had an Eastern Cooperative Oncology Group Performance Status of 0‐2 and a life expectancy of greater than 3 months. All patients provided signed, written informed consent. Patients were excluded if they had participated in a study in the 6 months before screening that involved administration of ^14^C. Other exclusion criteria included the presence of central nervous system or leptomeningeal metastasis, a prior malignancy other than the malignancies under study, cardiovascular impairment, active infection requiring systemic treatment, patients who were immunocompromised, or gastrointestinal issues that may impair the bioavailability of tazemetostat.

### Study Objectives

Part A of the study was designed to determine the plasma PK of [^14^C]‐tazemetostat, tazemetostat, and its metabolite EPZ‐6930, and to estimate tazemetostat absolute bioavailability. Part B of the study was designed to determine the total recovery and relative excretion of the administered radioactive dose in urine and feces; generate samples for characterizing and quantifying the metabolic profile of tazemetostat in plasma, urine, and feces; compare the total radioactivity concentration–time profiles in blood and plasma; and determine the plasma PK parameters of tazemetostat and EPZ‐6930.

### Sample Size

The sample size was determined on the basis of feasibility considerations in patients with advanced cancer. A total of 3 evaluable patients was adequate to describe the routes of excretion of tazemetostat and metabolites and to estimate the absolute bioavailability of tazemetostat.

### Sample Collection

For Part A, serial blood samples for the analysis of plasma concentrations of nonradiolabeled tazemetostat and [^14^C]‐labeled tazemetostat were collected over 24 hours after the morning oral dose of tazemetostat tablets on Day 15. For Part B, blood, plasma, urine, and fecal samples for analysis of total radioactivity and/or metabolite radioprofiling were collected for up to 120 hours after administration of the oral [^14^C]‐tazemetostat dose on Day 16. A full description of the sample collection can be found in Table S1.

### Sample Analysis

Plasma concentrations of [^14^C]‐tazemetostat were determined using high‐performance liquid chromatography–accelerator mass spectrometry. The validated lower limit of quantification was 0.816 pg/mL in neat plasma. Plasma concentrations of tazemetostat and its metabolites (EPZ‐6438 and EPZ‐6930) were determined using liquid chromatography followed by tandem mass spectrometry. The validated lower limit of quantification of the plasma assay for tazemetostat and EPZ‐6930 was 1.00 ng/mL in neat plasma. Radioactivity content was analyzed in plasma and urine using liquid scintillation counting, and in whole blood and feces by combustion analysis followed by liquid scintillation counting. The limits of detection of radioactivity content in these samples were 30.9‐39.1 ng equiv/mL in plasma, 26.0‐32.1 ng equiv/g in urine, 78.4‐119 ng equiv/mL in blood, and 161 ng equiv/g or greater in feces. See the Supplemental Methods for further details on sample preparation.

### Pharmacokinetic Analysis and Metabolic Profiling

Plasma concentration–time data for both tazemetostat (unlabeled and [^14^C]‐labeled) and EPZ‐6930 were analyzed by noncompartmental analysis using Phoenix WinNonlin (Phoenix WinNonlin Professional version 7.0 or later; PharSight Corp.).

PK outcome measures for [^14^C]‐tazemetostat, tazemetostat, and its metabolite EPZ‐6930 in blood and plasma after intravenous or oral administration of [^14^C]‐tazemetostat included the C_max_; time at which the maximum plasma concentrations were observed; observed terminal half‐life calculated as 0.6932/terminal elimination rate constant (estimated by linear regression through at least three data points in the terminal phase of the log concentration–time profile); AUC from time zero to 12 hours after dosing (AUC_0‐12_), AUC from time zero to the last quantifiable concentration (AUC_0‐t_), and AUC from time zero extrapolated to infinity (AUC_0‐∞_), using a linear up–log down trapezoidal rule, clearance, and volume of distribution following intravenous administration. The absolute bioavailability of tazemetostat was calculated as the dose‐normalized ratio of tazemetostat AUC_0‐12_ after oral tablet administration and [^14^C]‐tazemetostat AUC_0‐∞_ after the intravenous microdose on Day 15.

The total recovery and relative excretion of radioactivity in urine and feces were determined by comparing the total radioactivity detected in samples before and after an oral dose of [^14^C]‐tazemetostat. See the Supplemental Methods for further details on metabolite profiling and determination of radioactivity.

### Safety Assessment

Safety was continually assessed by monitoring adverse events (AEs) and vital signs, electrocardiography, and clinical laboratory tests. AEs were coded using the Medical Dictionary for Regulatory Activities Version 18.1.

## Results

### Patient Population

Between May 30 and August 6, 2018, 3 patients were enrolled (1 woman and 2 men) with a median age of 69 years (range, 53‐70 years) and mean weight of 75.3 kg (range, 60.0‐78.3 kg). All patients were White. All patients had a baseline Eastern Cooperative Oncology Group Performance Status of 1. Two patients had histologically or cytologically confirmed follicular lymphoma and 1 had diffuse large B‐cell lymphoma. All patients had received at least 3 prior lines of systemic anticancer therapy.

### Pharmacokinetic Assessment

Plasma PK parameters of [^14^C]‐labeled and unlabeled tazemetostat and EPZ‐6930 are shown in Table 1.

**Table 1:** Plasma PK Parameters of Tazemetostat, [^14^C]‐Tazemetostat, and EPZ‐6930

	Tazemetostat		EPZ‐6930		[^14^C]‐tazemetostat
PK parameter	Day 15 (oral tazemetostat tablets, 800 mg twice daily)	Day 16 (oral [^14^C]‐tazemetostat solution, 800 mg)	Day 15 (oral tazemetostat tablets, 800 mg twice daily)	Day 16 (oral [^14^C]‐tazemetostat solution, 800 mg)	Day 15 ([^14^C]‐tazemetostat, 12 µg intravenous)
C_max_, ng/mL (SD)^a^, ^b^	1180 (441)	1960 (1010)	1710 (428)	2230 (565)	707 (109)
Median t_max_, hours (range)	1.05 (1.02‐2.92)	0.917 (0.583‐0.967)	1.05 (1.02‐4.92)	1.97 (0.917‐1.98)	0.0167 (0.0167‐0.0333)
AUC_0‐last_, ng•h/mL (SD)^a^, ^b^	5270 (3900)	7940 (5220)	10,800 (5910)	14,200 (6650)	215 (59.1)
AUC_0‐∞,_ng•h/mL (SD)^a^, ^b^	5930 (4110)	8690 (5640)	12,900 (6070)	16,200 (8170)	223 (58.9)
AUC_0‐12_, ng•h/mL (SD)^a^, ^b^	5390 (3750)	7940 (5210)	11,200 (5380)	14,200 (6630)	209 (45.4)
F, % (SD)^a^	NA	NA	NA	NA	33.9 (12.2)
t_1/2_, hours (SD)^a^, ^b^	3.54 (0.439)	3.71 (0.762)	3.94 (0.618)	3.54 (0.318)	3.39 (23.8)
CL, L/h (SD)^a^	ND	ND	ND	ND	56.1 (13)
V_z_, L (SD)^a^	ND	ND	ND	ND	271 (33.8)
MPR (SD)^a^	NA	NA	2.39 (0.461)	2.02 (0.336)	NA

AUC_0‐∞_, area under the concentration–time curve from time zero extrapolated to infinity; AUC_0‐12_, area under the concentration–time curve from time zero to 12 hours after dosing; AUC_0‐t_, area under the concentration–time curve from time zero to the last quantifiable concentration; CL, clearance; C_max_, maximum observed plasma concentration; CV, coefficient of variation; F, absolute bioavailability; MPR, metabolite‐to‐parent ratio; NA, not applicable; ND, not determined; PK, pharmacokinetic; t_1/2_, terminal half‐life; t_max_, time to reach maximum observed plasma concentration; V_z_, volume of distribution.

^a^ Data reported as arithmetic mean (SD).

^b^ Units for Day 15 (intravenous [^14^C]‐tazemetostat bolus, 12 µg) are pg/mL for C_max_ and h·pg/mL for AUC measures.

**Figure 2:**
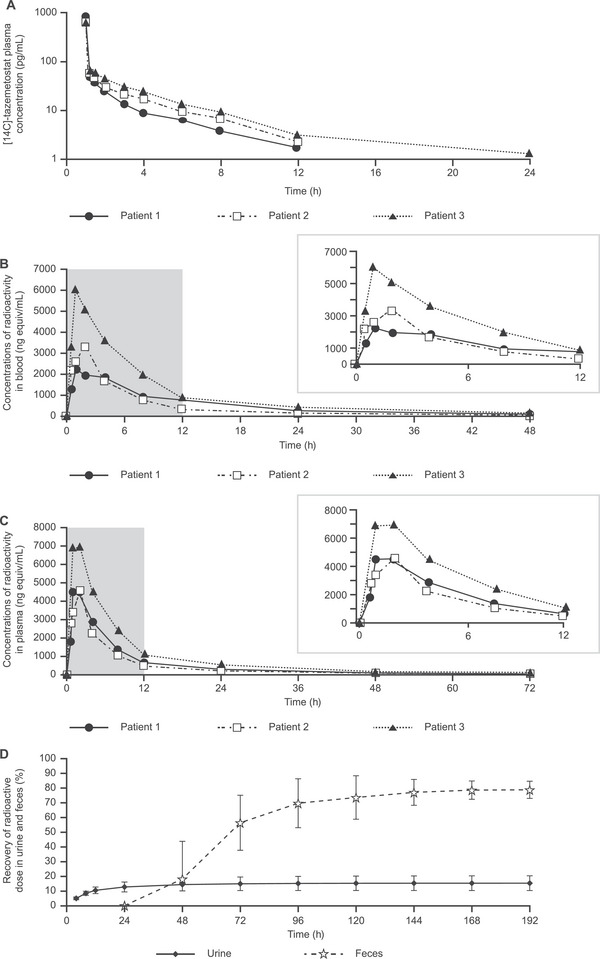
Concentration of radioactivity following an 800‐mg oral dose and 12‐µg intravenous microdose of [^14^C]‐tazemetostat in blood, plasma, urine, and feces. (A) [^14^C]‐tazemetostat plasma concentrations following a 12‐µg intravenous microdose. (B) Concentrations of total radioactivity in blood following 800 mg of oral [^14^C]‐tazemetostat. (C) Concentrations of total radioactivity in plasma following 800 mg of oral [^14^C]‐tazemetostat. (D) Mean (SD) cumulative recovery of total radioactivity in urine and feces following 800 mg of oral [^14^C]‐tazemetostat. Patient 1 and Patient 2 had met the exit criteria by 120 hours of >90% accumulated recovery. Therefore, excreta collections were stopped; their mean cumulative urine and feces recoveries have been extrapolated to 192 hours, replicating the 120 recoveries, to allow overall mean values to be plotted after 120 hours. SD, standard deviation.

Orally administered tazemetostat 800‐mg twice‐daily tablets on Day 15 were rapidly absorbed. Based on tazemetostat AUC_0‐12_ as a tablet and AUC_0‐∞_ as an intravenous bolus, the mean absolute bioavailability was 33.9%. Plasma concentrations of [^14^C]‐tazemetostat for up to 24 hours following a 12 µg intravenous microdose on Day 15 are shown in Figure 2A.

Following administration of tazemetostat oral solution on Day 16, the arithmetic mean C_max_ was 1.67‐ and 1.30‐fold higher for tazemetostat and EPZ‐6930, respectively, than observed on Day 15. The arithmetic mean AUC_0‐12_ was 1.47‐fold and 1.27‐fold higher on Day 16 than Day 15 for tazemetostat and EPZ‐6930, respectively. Similar trends were observed with AUC_0‐t_ and AUC_0‐∞_.

### Metabolite and Radioprofiling

**Figure 3:**
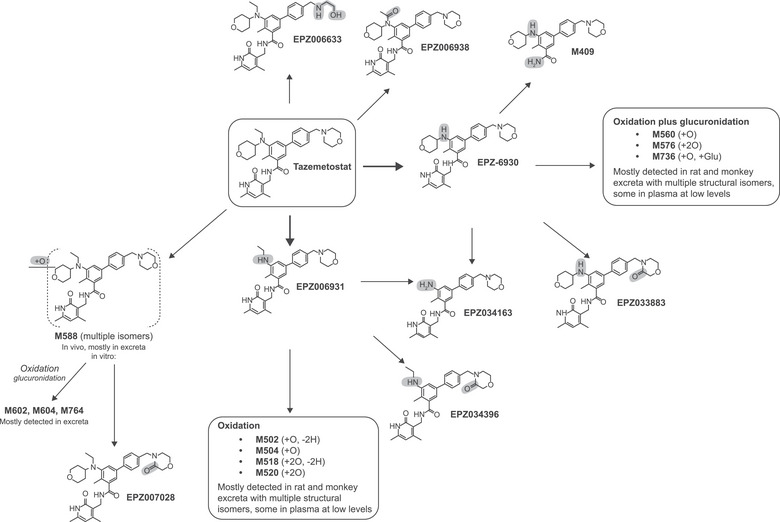
Metabolic profiling of tazemetostat.

Figure 3 presents a summary of tazemetostat's metabolite profile. Tazemetostat and up to 34 metabolites were detected across the plasma samples analyzed. Unchanged tazemetostat accounted for 22.4% of the total drug‐related exposure. The most notable plasma metabolites were 2 products of N‐dealkylation reactions, EPZ‐6930 and EPZ006931, and a product of further *N*‐dealkylation of EPZ‐6930, EPZ034163. EPZ‐6930, EPZ006931, and EPZ034163 accounted for 31.8%, 11.0%, and 3.5% of the total drug‐related exposure, respectively. All other metabolites in plasma represented less than 2% of the total exposure (Table 2).

**Table 2:** Metabolite Profiling Summary Following [^14^C]‐Tazemetostat 800‐mg Oral Solution

Molecule	Plasma total drug‐related exposure, %	Urine total dose, %	Feces total dose, %
Tazemetostat	22.4	1.4	ND
*N*‐detrimethylpyridinone + *N*‐detetrahydropyran product of tazemetostat and *N*‐detrimethylpyridinone product of tazemetostat	2.5^a^	ND	ND
EPZ034163	3.5	ND	ND
EPZ‐6930	31.8	6.7	7.5^a^, ^b^
EPZ006633 and EPZ‐6930	ND	ND	
EPZ‐6930 and EPZ006931	ND	ND	3.41^a^, ^b^
EPZ006931	11.0	ND	
Monooxygenation product of EPZ‐6930	ND	ND	5.8
*N*‐detetrahydropyran product of tazemetostat + monooxidation	ND	ND	7.0
Monooxygenation product of EPZ‐6930 and the monooxygenation product of tazemetostat	ND	ND	6.2^a^
Dioxidation product of EPZ‐6930	ND	ND	4.9
*N*‐detetrahydropyran product of tazemetostat and oxidation	ND	ND	3.0
*N*‐desethyl EPZ006633 and dioxidation product of tazemetostat	ND	ND	8.8^a^

Reported values are means.

ND, not detected.

^a^ Co‐eluting metabolites (1 radiochromatogram peak contained 2 co‐eluting metabolites).

^b^ In feces, radiochromatograms EPZ‐6930, EPZ006633 and EPZ006931 partially co‐elute.

Excretion via urine was a minor route of elimination, with less than 20% of the total administered radioactive dose being excreted via urine. Unchanged tazemetostat was a minor component of the urine profiles, representing only 1.4% of the total dose. Up to 57 metabolites were detected in urine. However, only EPZ‐6930 accounted for more than 1% of the total dose and represented 6.7% of the dose excreted in urine. All other metabolites in the urine represented less than 1% of the total radioactive dose and therefore were considered minor components and at levels too low for identification.

Excretion via feces was the major route of excretion, accounting for 67%‐84% of the total administered radioactive dose. Unchanged tazemetostat was not detected but up to 35 potential metabolites were identified. However, all regions of radioactivity detected in the radioprofiles of the pooled feces samples accounted for less than 10% of the total administered dose. Many of these regions were found to contain 2 co‐eluting metabolites. The notable metabolites in feces (accounting for 3%‐9% of the dose) were EPZ‐6930, EPZ006633, EPZ006931, monooxygenation product of EPZ‐6930, *N*‐detetrahydropyran product of tazemetostat mono‐oxidation, mono‐oxygenation product of EPZ‐6930, mono‐oxygenation product of tazemetostat (M588_1), dioxidation product of EPZ‐6930 (M576_3), *N*‐detetrahydropyran product of tazemetostat plus oxidation (M520), *N*‐desethyl EPZ006633 (M518_1), and dioxidation product of tazemetostat (M604_5). All other metabolites detected in the feces’ profiles accounted for less than 3% of the total administered dose. Representative radiochromatograms for plasma, feces, and urine following oral administration of [^14^C]‐tazemetostat can be found in Figure S2.

### Mass Balance

Following oral administration of [^14^C]‐tazemetostat, radioactivity was rapidly absorbed and detected in all patients 30 minutes after dosing. The mean tazemetostat concentrations were 2250 ng equiv/mL in blood and 2930 ng equiv/mL in plasma. Maximum concentrations of total radioactivity were reached 1‐2 hours after dosing in blood and 2 hours in plasma. Radioactivity was no longer detected in blood by 72 hours after dosing, with 1 patient having no detectable radioactivity at 48 hours after dosing. However, radioactivity was still detected in plasma for all patients at 72 hours after dosing. The quantification differences in radioactivity between blood and plasma were possibly due to differences in the limits of detection. Over 0.5‐72 hours after dosing, mean concentrations of drug‐related material in blood were consistently lower than those in plasma, with mean blood:plasma ratios ranging from 0.63 to 0.75. This suggests that there was no preferential distribution of drug‐related material into red blood cells. Concentrations of total radioactivity in blood and plasma following 800 mg of oral [^14^C]‐tazemetostat are shown in Figure 2B and C, respectively.

Total recoveries of radiolabeled material ranged from 93.2% to 94.7%. Most excreted dose was recovered within 48 hours in urine and by 96 hours in feces, in 2 of the 3 patients. Radioactivity in feces was detected for up to 120 hours after dosing for 2 patients and up to 192 hours after dosing for 1 patient. Fecal elimination represented the principal route of elimination with a mean of 78.9% ± 5.8% of the administered dose. Renal excretion accounted for a mean of 15.4% ± 5.0% of the administered dose. The cumulative recovery of total radioactivity in urine and feces following 800 mg of oral [^14^C]‐tazemetostat is presented in Figure 2D.

### Safety

All 3 patients experienced at least 1 treatment‐related treatment‐emergent AE (TEAE) and experienced at least 1 serious TEAE, none of which resulted in a dose reduction or interruption. Two patients experienced at least 1 Grade 3 or 4 treatment‐related TEAE. One patient experienced Grade 3 cellulitis that was considered possibly related to tazemetostat. Another patient experienced Grade 4 neutropenic sepsis that was considered possibly related to tazemetostat. Overall, the nature and severity of TEAEs observed were as expected and consistent with the known safety profile of tazemetostat.

## Discussion

This study was conducted to characterize the PK, determine the absolute bioavailability, characterize the disposition and metabolism of tazemetostat, and investigate the metabolic profile of tazemetostat of an intravenous microdose of [^14^C]‐labeled and oral unlabeled and [^14^C]‐labeled tazemetostat in patients with B‐cell lymphomas or advanced solid tumors.

Plasma concentrations of tazemetostat and EPZ‐6930 were observed up to 12 hours for 2 patients, with 1 patient having quantifiable levels for up to 24 hours after dosing for [^14^C]‐tazemetostat as an intravenous bolus and oral solution, respectively. Maximum concentrations of oral or intravenous tazemetostat were reached within 2 hours in the blood and plasma. Higher C_max_ and AUC were observed in tazemetostat given as an oral solution on Day 16 compared with when administered as tablets on Day 15. This may be a result of increased absorption associated with the liquid formulation. The terminal half‐life for tazemetostat and EPZ‐6930 was between 3 and 4 hours, regardless of the formulation or route of administration.

Following oral administration, tazemetostat was extensively metabolized, with several potential metabolites detected in plasma, urine, and feces. The most predominant metabolites, EPZ‐6930 and EPZ006931, were products of 2 *N*‐dealkylation reactions at the aniline nitrogen of tazemetostat. The EPZ‐6930:tazemetostat ratio was approximately 2‐fold based on the AUC. Tazemetostat also underwent dealkylation and oxidation to form less abundant metabolites. The formation of the 2 major inactive metabolites, EPZ‐6930 and EPZ006931, is owed to hepatic metabolism by CYP3A. EPZ‐6930 was further metabolized by CYP3A to form its oxygenated products.

Fecal excretion was the primary route of elimination for oral [^14^C]‐tazemetostat, with urine elimination accounting for a significantly lower proportion of recovery of the administered dose. Large interpatient variability was observed in the recovery of [^14^C]‐tazemetostat in urine and fecal samples. The high coefficient of variation was due to heterogeneity of the data as a result of interpatient variability, small sample size and the need to extrapolate to 192 hours due to patients meeting the exit criteria. Although the conventional size of this type of PK study is 4‐6 subjects, there was a need to balance the burden on the patients, given the advanced stage of cancer of the participants enrolled. Therefore, it was determined that 3 was an adequate population size for this drug and provided sufficient data to characterize its absorption, distribution, metabolism, and excretion profile.

## Conflicts of Interest

Yingxue Chen and Julien Ogier are employees of Ipsen. Renli Teng is a partner of CareCeutics LLC, contracted by Ipsen.

## Funding

This study was sponsored by Epizyme, Inc., An Ipsen company.

## Supporting information



Supporting Information

Supporting Information
