# Supplementary material for: Bioavailability, Metabolism, and Excretion of [14C]‐Tazemetostat in Patients With B‐Cell Lymphomas or Advanced Solid Tumors
Source: Clin Pharmacol Drug Dev. 2025 Feb 3;14(3):231–9. doi: 10.1002/cpdd.1508 (PMC11905873; doi:10.1002/cpdd.1508)
Supplement: Supplementary file 1 — Supporting Information [file CPDD-14-231-s002.docx]

**Supplementary materials for: Chen Y, et al. Bioavailability, Metabolism, and Excretion of [^14^C]-Tazemetostat in Patients With B-cell Lymphomas or Advanced Solid Tumors**

**Contents**

[**Supplementary Methods** 2](#_Toc174618246)

[Sample preparation for liquid scintillation counting 2](#_Toc174618247)

[Determination of radioactivity by liquid scintillation counting 2](#_Toc174618248)

[Metabolite profiling by high resolution mass spectrometry 3](#_Toc174618249)

[**Supplementary Table 1. Description of biological sample collection for Parts A and B** 4](#_Toc174618250)

[**Supplementary Figure 1. Structure of [^14^C]-tazemetostat** 7](#_Toc174618251)

[**Supplementary Figure 2. Representative radio-chromatograms for plasma (A–E), urine (F), and feces (G) following oral administration of [14C]-tazemetostat 800 mg** 8](#_Toc174618252)

**Supplementary Methods**

## Sample preparation for liquid scintillation counting

For plasma samples, 2 mL was vortex mixed with approximately 3 volumes of acetonitrile (6 mL) for 2 minutes and then centrifuged (3000 rpm, 10 minutes, 4°C). The resulting supernatant was transferred to a vessel, and evaporated to near-dryness under a stream of nitrogen gas at a temperature of 30°C. The residue was reconstituted in a suitable volume (600 µL) of water:acetonitrile solution (4:1, v/v). The recovery of radioactivity in the final extracts was determined by liquid scintillation counting.

For fecal samples, 2 g was vortex mixed for approximately 2 minutes with approximately 0.5 volume of methanol (1 mL), 3 volumes of acetonitrile (6 mL) were then added. The sample was then centrifuged, (3000 rpm, 10 minutes, 4°C), and the resulting supernatant (Extract 1) transferred to a vessel. The pellet was extracted again as described above (Extract 2). Both extracts were combined, and evaporated to near-dryness under a stream of nitrogen gas at a temperature of approximately 30˚C. The residue was reconstituted in 2 mL of water/acetonitrile (4:1, v/v) solution (reconstitution 1). The extracts were centrifuged (13000 rpm, 10 minutes, 4°C), to remove particulate matter. A 0.5 mL aliquot of each concentrated sample extract was evaporated to near-dryness under a stream of nitrogen gas at a temperature of 30°C. The residue was reconstituted (reconstitution 2) in a suitable volume of mobile phase A (25 mM ammonium formate [aqueous] pH 3.5) and then centrifuged (13000 rpm, 10 minutes, 4°C), to remove particulate matter. The recovery of radioactivity in the concentrated extracts was determined by liquid scintillation counting.

Pooled urine samples were directly injected for analysis without any pre-treatment.

## Determination of radioactivity by liquid scintillation counting

Radioactivity in all samples was measured for 5 minutes using a Packard Tri-Carb liquid scintillation counter Model 2100TR, 2900TR or 3100TR (Canberra Packard, Pangbourne, Berks) with the facilities for computing quench-corrected disintegrations per minute (dpm). Efficiency correlation curves were prepared and routinely checked by the use of [^14^C]-toluene or Ultima Gold™ quenched standards (supplied by PerkinElmer LAS [UK] Ltd).

The limit of quantification for each batch of samples analyzed by direct counting was taken as twice the mean background disintegration rate obtained from vials containing liquid scintillant. If a mean original count was below the limit of quantification, the count was defined as not detectable.

The limit of quantification of each batch of whole blood samples and feces analysed by combustion was taken as twice the mean background disintegration rate obtained when Combusto-Cones™ containing Combusto-Pads were combusted.

## Metabolite profiling by high resolution mass spectrometry

Analysis was performed with a Q Exactive Plus mass spectrometer (Thermo Scientific, UK) connected to a Nexera X2 DGU-20A5R (Shimadzu, UK) liquid chromatography system. For metabolite profiling, the column eluent from the liquid chromatography system was diverted for parallel mass spectrometry and radio-detection. Off-line radio-detection was used for plasma, faeces and urine. Offline radio-detection was performed using a HTC PAL fraction collector (CTC Analytics AG, Switzerland) with TopCount NXT (Perkin Elmer LAS (UK) Ltd). Measurements were performed using fraction collection at 10 second intervals into 96-well Lumaplates. The plates were then dried, sealed and the fractions counted for 2 minutes per well. Data were captured with Laura software (LabLogic Systems Ltd).

**Supplementary Table 1. Description of biological sample collection for Parts A and B**

| **Sample** | **Part A** | **Part B** |
| --- | --- | --- |
| **Blood** | **Unlabeled tazemetostat and EPZ-6930 plasma concentrations**  1.5 mL serial blood samples collected after the oral dose of tazemetostat on Day 15 at: pre-dose (within 30 minutes prior to the oral dose), and 0.5, 1 (end of IV bolus), 2, 3, 4, 5, 6, 8, and 12 hours after administration of the morning oral dose of tazemetostat 800 mg  **[^14^C]-labeled-tazemetostat plasma concentrations**  4 mL serial blood samples collected at: pre-dose (within 30 minutes prior to the oral dose) and 1 (end of IV bolus), 1.25, 1.5, 2, 3, 4, 6, 8, and 12 hours after administration of the morning oral dose of tazemetostat 800 mg | **Plasma [^14^C]-tazemetostat concentration**  4 mL blood sample collected immediately prior to administration of [^14^C]-tazemetostat oral solution (24 hours after the morning oral dose on Day 15)  **Tazemetostat and EPZ-6930 plasma concentrations**  1.5 mL serial blood samples collected at: pre-dose (within 30 minutes prior to the oral dose), and 0.5, 1, 2, 4, 6, 8, and 12 hours after administration of tazemetostat as the oral solution  **Plasma and blood radioactivity concentrations**  Serial 6 mL blood samples collected at: pre-dose (within 30 minutes prior to the oral dose), and 0.5, 1, 2, 4, 8, 12, 24, 48, and 72 hours after administration of tazemetostat oral solution  **Tazemetostat metabolites in plasma**  Serial 10 mL blood samples collected at: 1, 2, 4, 8, and 12 hours after administration of tazemetostat oral solution |
| **Urine** | Not collected | Patients were asked to empty their bladder immediately prior to administration of [^14^C]-tazemetostat on Day 16  Total urine output was collected over the intervals of 0–4, 4–8, 8–12, and 12–24 hours after administration of the morning dose on Day 16, then over 24-hour intervals on subsequent days until the patient was discharged  Complete daily urine output could be collected at home if ≥90% of the radioactive dose had not recovered before discharge |
| **Feces** | Not collected | Total fecal output was collected over 24-hour intervals from the time of the morning tazemetostat dose on Day 16 until the patient was discharged  Complete daily fecal output could be collected at home if ≥90% of the radioactive dose was not recovered before discharge |

Abbreviations: IV, intravenous.

**Supplementary Figure 1. Structure of [^14^C]-tazemetostat**

**
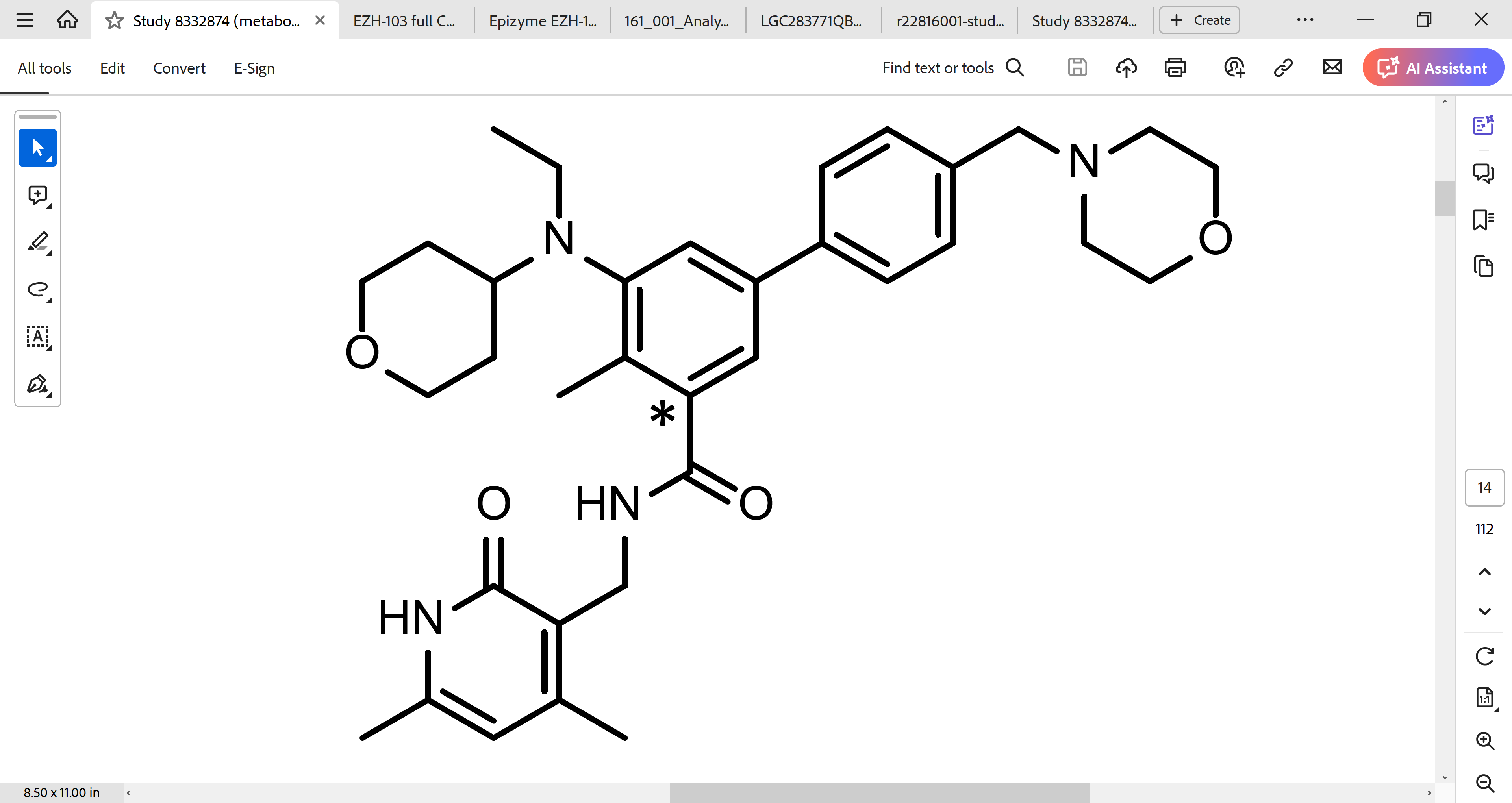
**

The position of ^14^C is marked by the asterisk.

**Supplementary Figure 2. Representative radio-chromatograms for plasma (A–E), urine (F), and feces (G) following oral administration of [^14^C]-tazemetostat 800 mg**


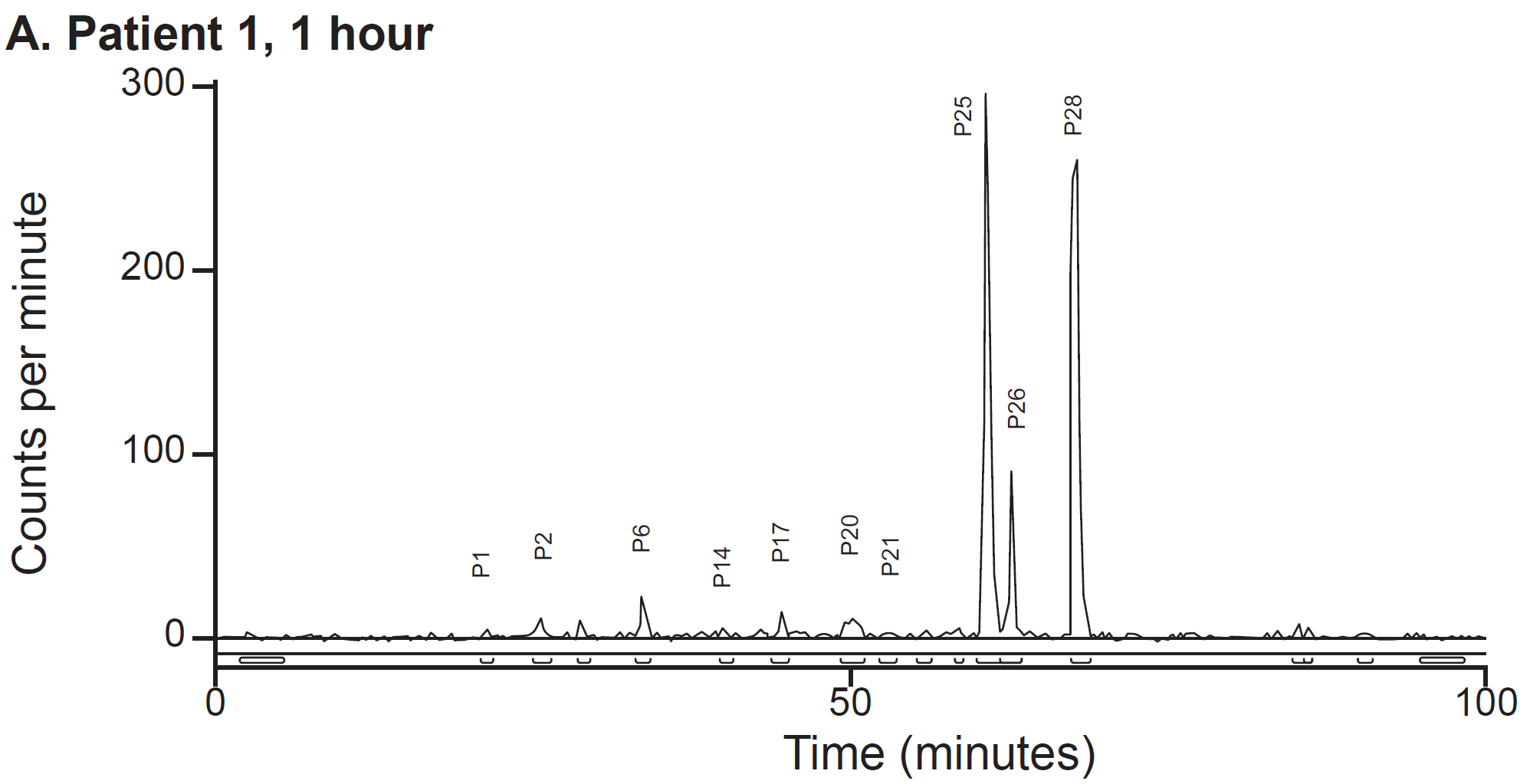


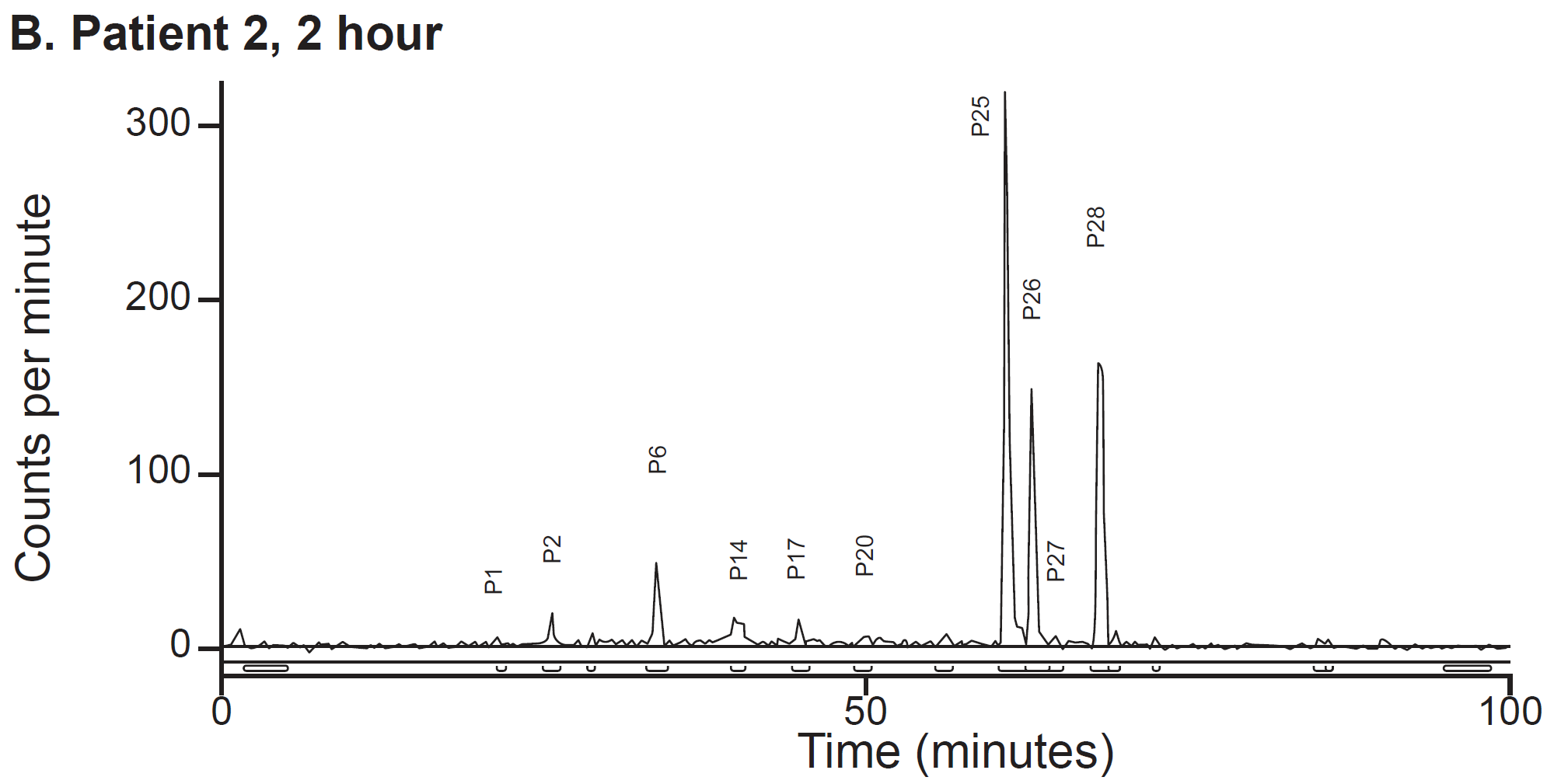


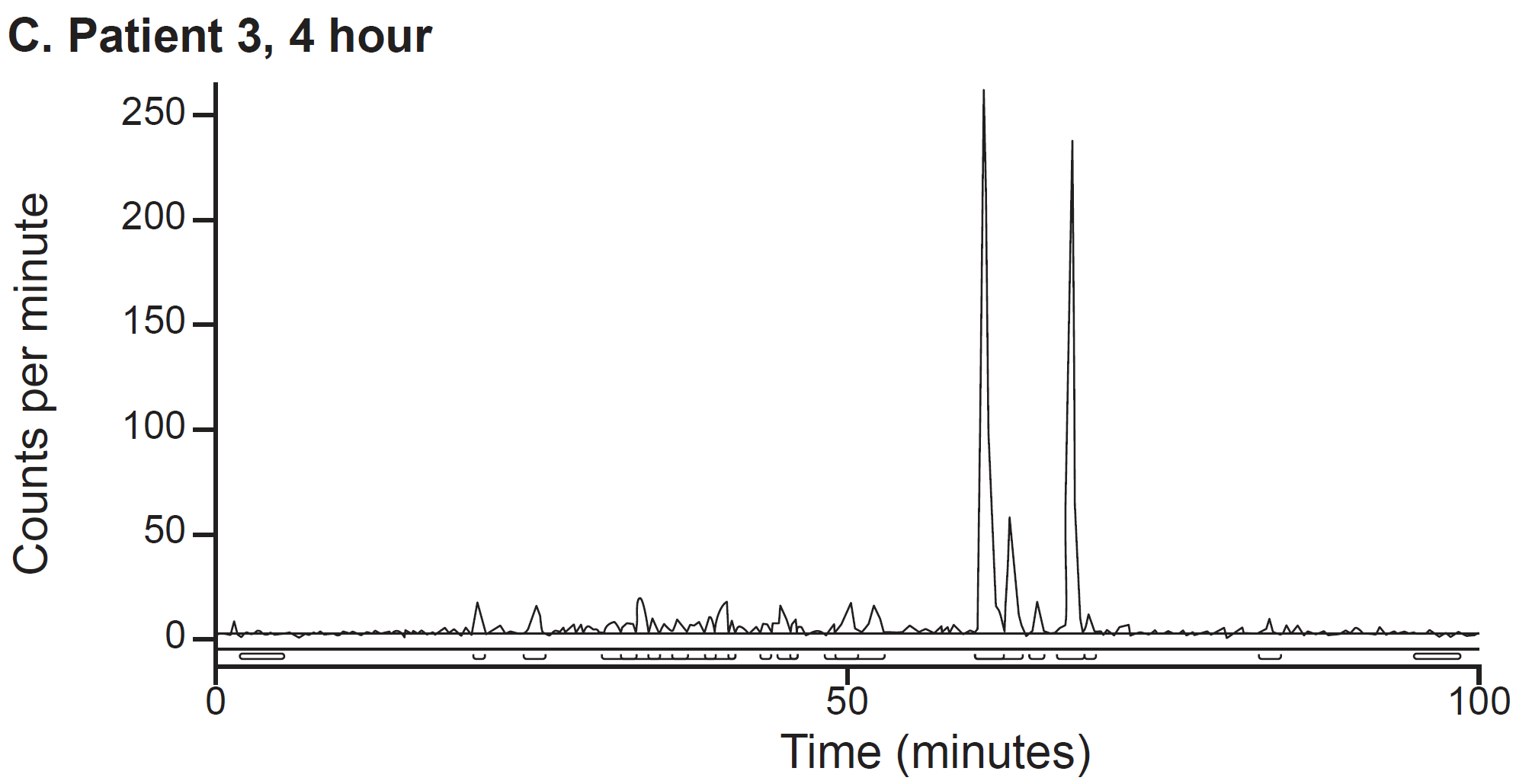


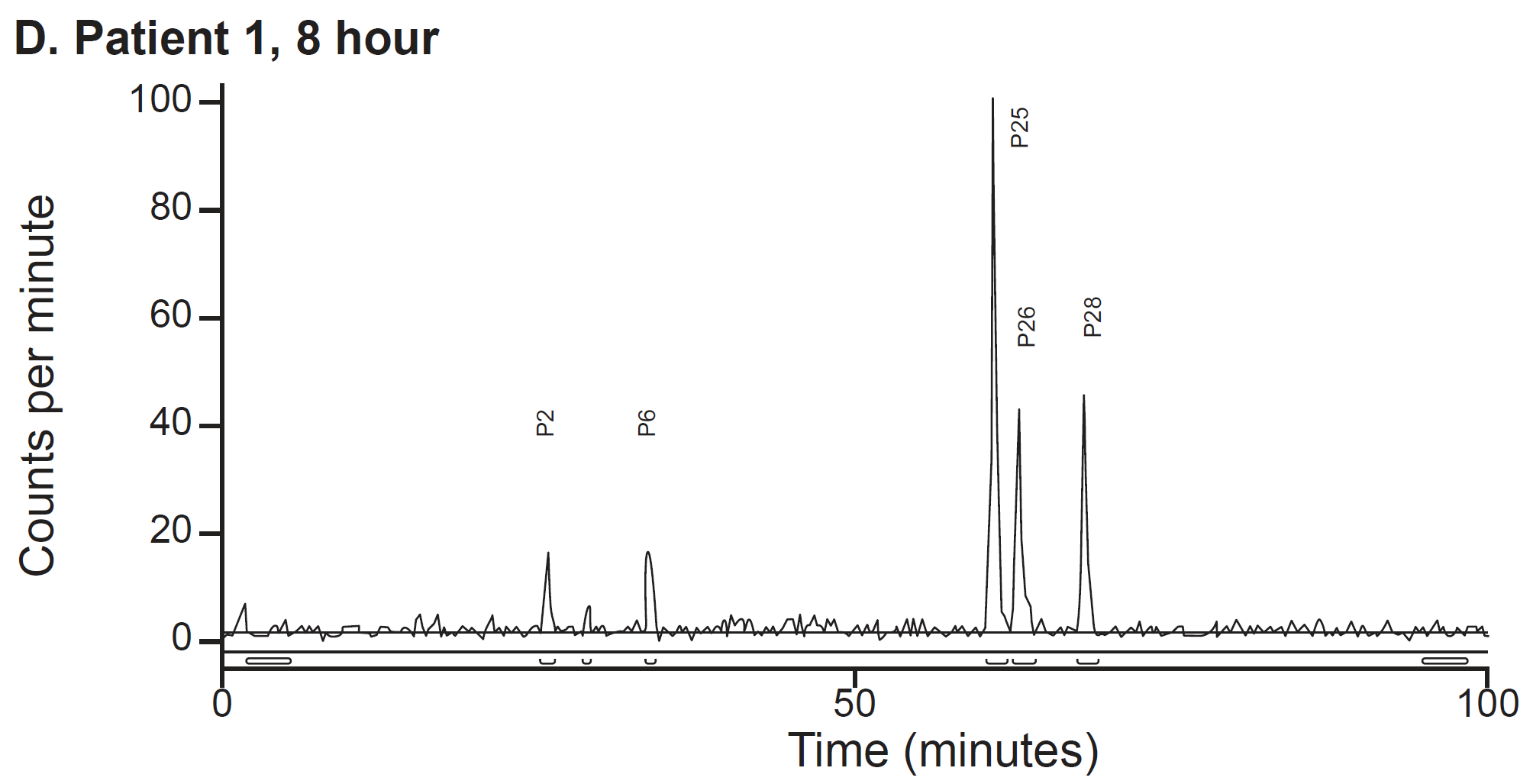


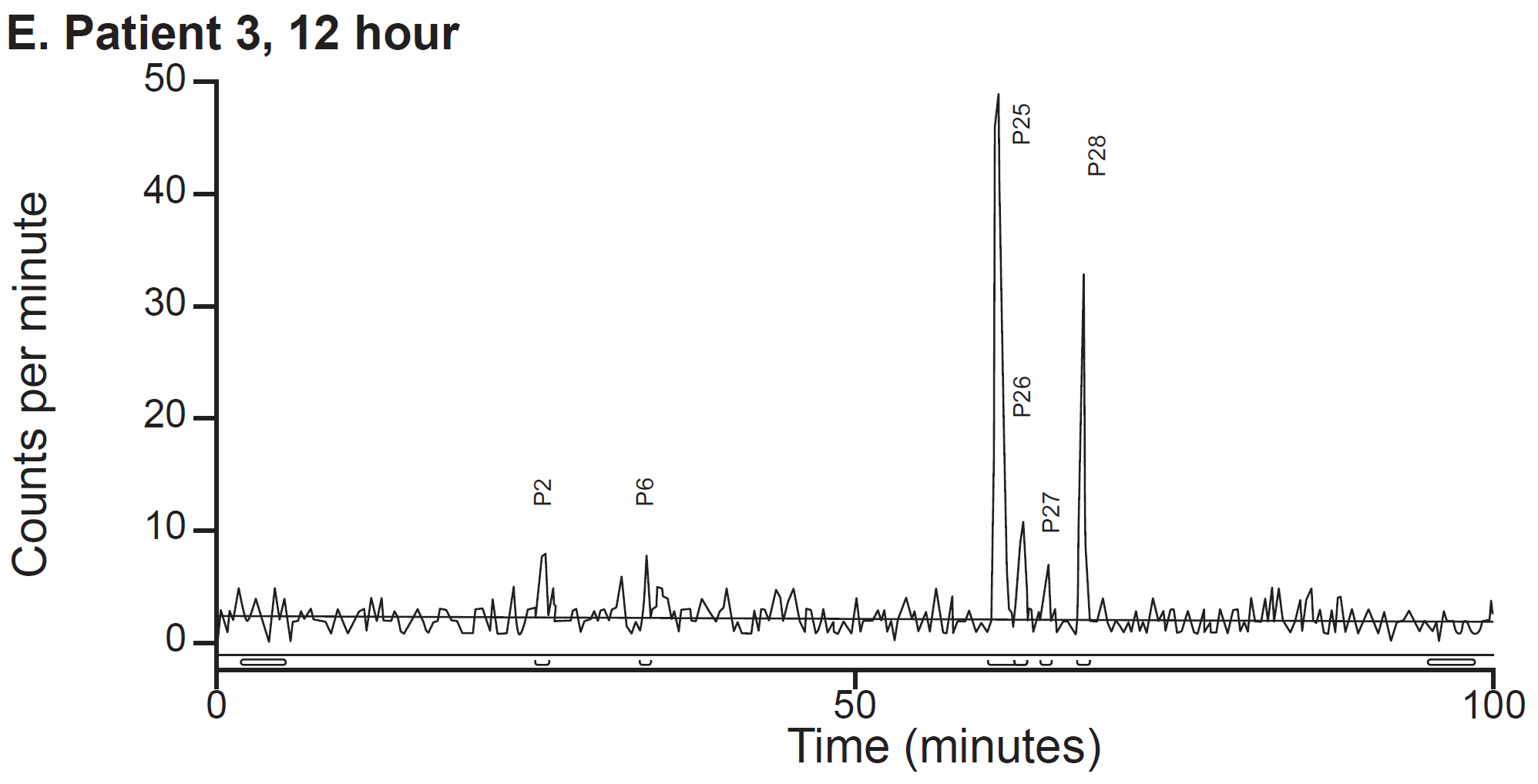


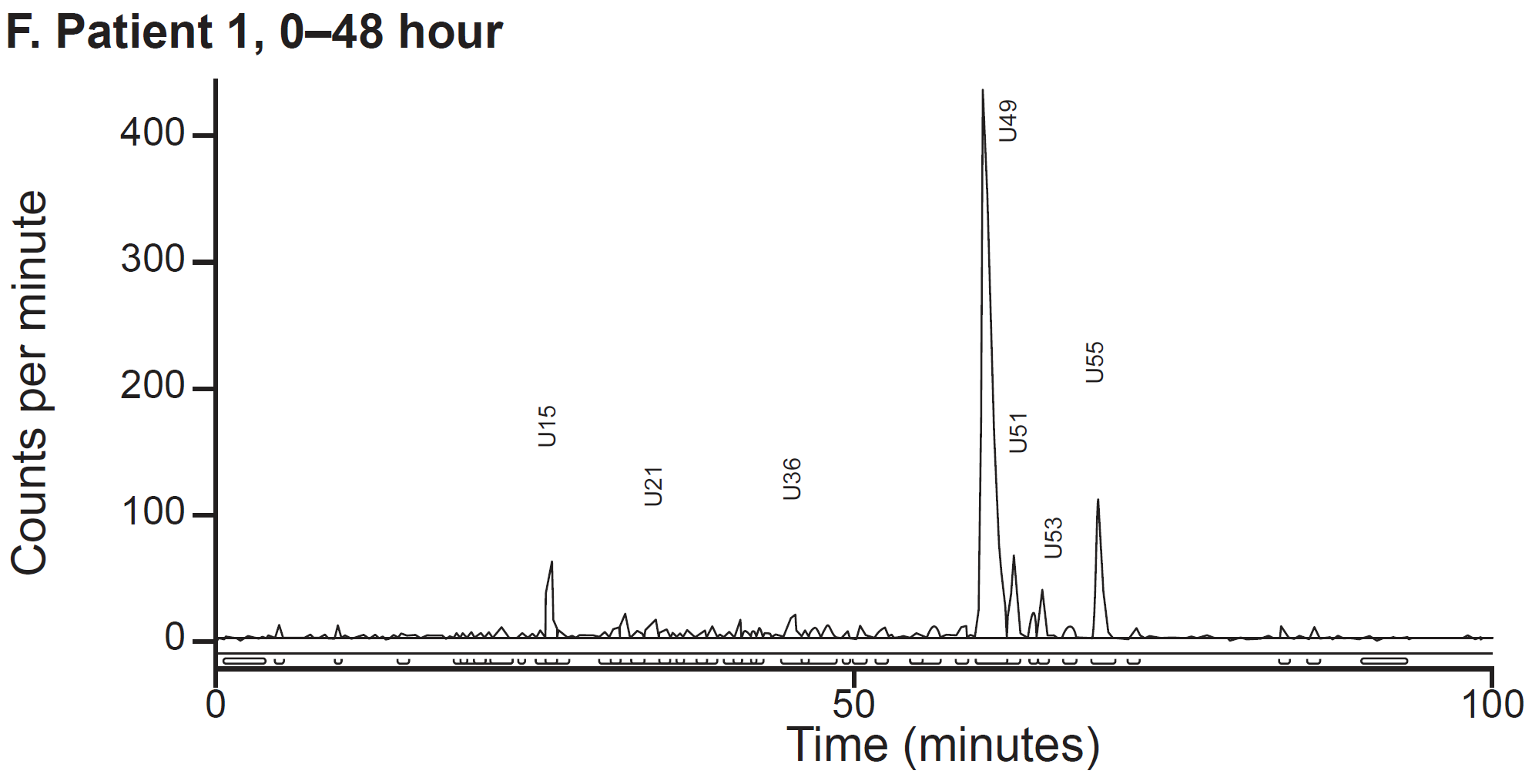


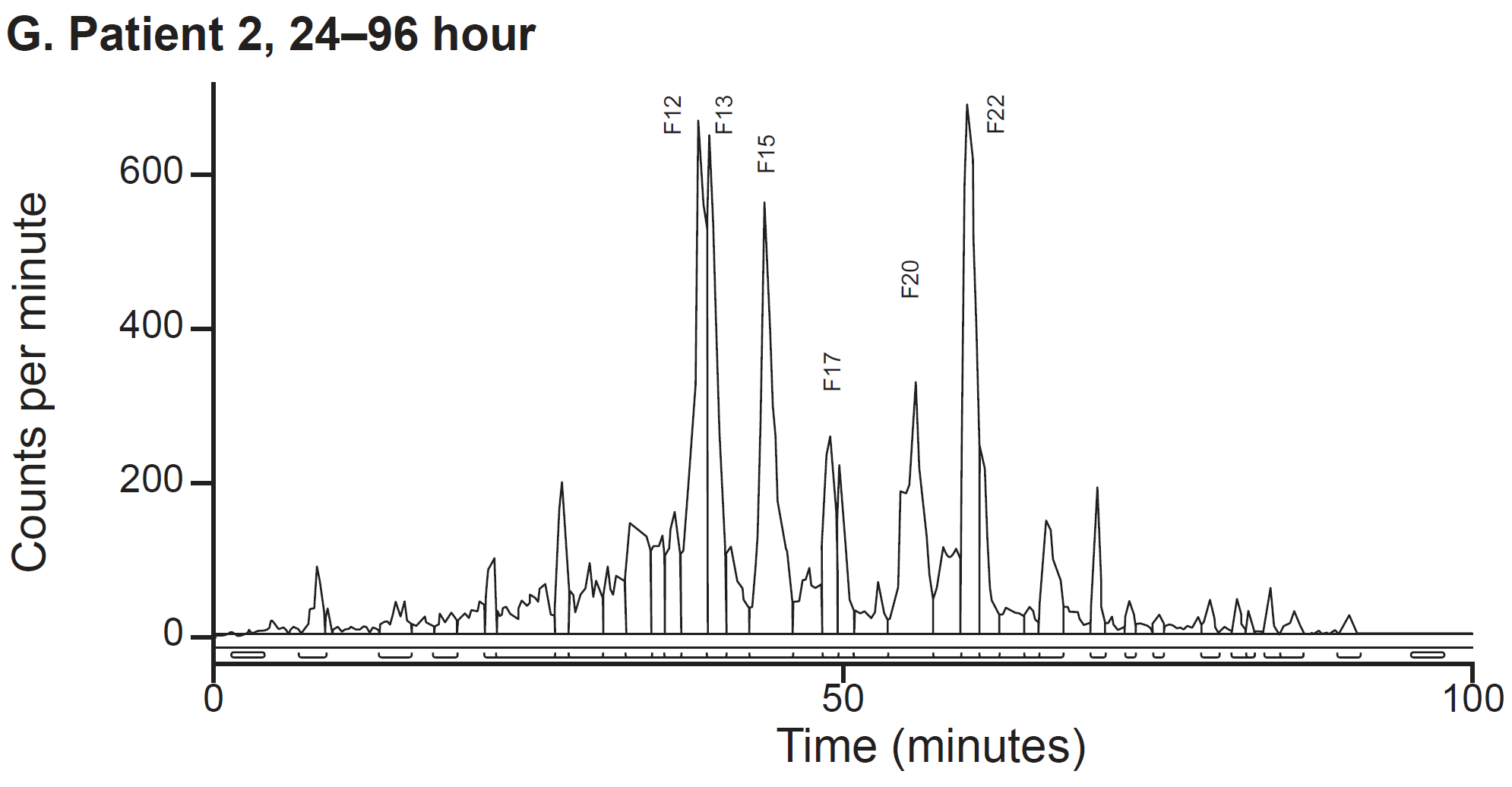


The molecules identified in the peaks in Supplementary Figure 2 are as follows: P2, *N*-detrimethylpyridinone and *N*-detetrahydropyran product of tazemetostat or N-detrimethylpyridinone product of tazemetostat; P6, EPZ034163; F12 (broad peak), monooxygenation product of EPZ-6930. F13, *N*-detetrahydropyran product of tazemetostat and monooxidation; F15, monooxygenation product of EPZ-6930; F15, dioxidation product of EPZ-6930; F17, *N*-detetrahydropyran product of tazemetostat and oxidation; F18, *N*-desethyl EPZ006633; F20, dioxidation product of tazemetostat; F22, EPZ006633; P25/F22 and F23 (broad peak)/U49, EPZ-6930; P26/F23, EPZ006931; P28/U55, tazemetostat. Molecules associated with undefined peaks were unidentified.

Abbreviations: F, feces; P, plasma; U, urine.
