# Supplementary material for: Bioavailability, Metabolism, and Excretion of [14C]‐Tazemetostat in Patients With B‐Cell Lymphomas or Advanced Solid Tumors
Source: Clin Pharmacol Drug Dev. 2025 Feb 3;14(3):231–9. doi: 10.1002/cpdd.1508 (PMC11905873; doi:10.1002/cpdd.1508)
Supplement: Supplementary file 2 — Supporting Information [file CPDD-14-231-s001.docx]

**Plain language summary for: Chen Y, et al. Bioavailability, Metabolism, and Excretion of [^14^C]-Tazemetostat in Patients With B-cell Lymphomas or Advanced Solid Tumors**

Tazemetostat is a medicine approved by the US Food and Drug Administration (FDA) for patients with specific cancers: follicular lymphoma (a type of white blood cell cancer) or epithelioid sarcoma (a rare cancer usually found in the soft tissues of the body, such as the arms or legs). The aim of this study was to find out how the body absorbs, processes, and gets rid of tazemetostat in patients with cancer.

To be able to detect the tazemetostat, patients were given two doses of radioactive tazemetostat. The radioactive doses make it easier for scientists to detect tazemetostat in blood, urine, and stool samples. For the first 14 days, patients received an 800 mg dose of non-radioactive tazemetostat as tablets twice a day. On Day 15, patients received an 800 mg dose of non-radioactive tazemetostat as tablets in the morning, followed 1 hour later by a small radioactive dose of tazemetostat as an injection into a vein. On the morning of Day 16, patients received a liquid containing radioactive tazemetostat. After 12 hours, patients received an 800 mg dose of non-radioactive tazemetostat as tablets and continued to take tazemetostat for as long as it was effective against their disease.

Three patients with blood cancer participated in the study. Tazemetostat was absorbed quickly into the blood, reaching a maximum amount within 1 hour. Based on the amount of radioactive tazemetostat detected, the average amount of tazemetostat that was taken into the blood and moved around blood vessels was 34%. Most tazemetostat was lost through the stool, with a smaller amount being removed in the urine. Most of the tazemetostat lost through the stool happened within 96 hours after the patients took the radioactive tazemetostat liquid. Some small amounts of tazemetostat were still detected up to 120 hours post-dose for two of the patients, and up to 192 hours for one patient. Most tazemetostat lost through the urine happened within 48 hours after the patients took the radioactive tazemetostat liquid.

Studies like this help researchers to understand how the body processes and removes medications and helps healthcare providers to appropriately dose medicines.
